# *MIB Guides*: Preclinical radiopharmaceutical dosimetry

**DOI:** 10.21203/rs.3.rs-3225362/v1

**Published:** 2023-08-14

**Authors:** Lukas M. Carter, Pat B. Zanzonico

**Affiliations:** Department of Medical Physics, Memorial Sloan Kettering Cancer Center, New York, NY, USA

## Abstract

Preclinical dosimetry is essential for guiding the design of animal radiopharmaceutical biodistribution, imaging, and therapy experiments, evaluating efficacy and/or toxicities in such experiments, ensuring compliance with ethical standards for animal research, and providing reasonable initial estimates of normal-organ doses in humans, required for clinical translation of new radiopharmaceuticals. This MIB guide provides a basic protocol for obtaining preclinical dosimetry estimates with organ-level dosimetry software.

## INTRODUCTION

Internal dosimetry entails the determination of the absorbed dose delivered to humans or animals from imaging as well as therapeutic radiopharmaceuticals. It thus serves as an “indicator” of the probability and/or severity of the radiation-related biological effects of administered radiopharmaceuticals. *Preclinical dosimetry* largely comprises two paradigms: 1) small-animal dosimetry, wherein rodent dose estimates are derived and 2) translational dosimetry, wherein prospective human dosimetry estimates are derived from animal biodistribution data. Several examples of the applications of these respective paradigms are described below.

### Small-animal dosimetry.

Small-animal dosimetry is valuable in assessing and managing radiation exposures to laboratory animals used in preclinical studies. For preclinical radiopharmaceutical therapy, dosimetry provides a meaningful basis on which to assess or predict efficacy, toxicities, and therapeutic ratios (e.g., the tumor-to-normal organ absorbed dose ratios). Frequently, it is employed in the design of radiopharmaceutical therapy studies to estimate the administered activities required to induce a demonstrable tumor response without exceeding tolerable doses for at-risk organs. The number of animals needed to characterize such radiopharmaceuticals can thereby be minimized while still yielding useful dose-response data. For small-animal biodistribution studies, including those utilizing PET, SPECT, and/or *ex vivo* gamma counting measurements, dosimetry can also help ensure the planned administered activity is unlikely to cause radiogenic toxicities that may perturb the study output. In particular, this can be important in experiments utilizing engineered mouse models that have deficient or absent DNA repair mechanisms and are therefore highly radiosensitive (e.g., SCID mice). Dosimetry is also valuable for ensuring compliance with ethical standards for animal research – for example, minimizing pain in experimental animals.

### Translational dosimetry.

The chief role of translational dosimetry is to satisfy regulatory requirements for clinical translation of new radiopharmaceuticals as well as to avoid excessively high doses in human research subjects or patients. The FDA and other regulatory bodies typically mandate (*de jure* or *de facto*) that human dosimetry estimates be extrapolated from animal models prior to human administration of radiopharmaceuticals; such estimates accompany translational mechanisms such as investigational new drug applications. Translational dosimetry estimates can also be valuable at the early stages of radiopharmaceutical development – for example, in identifying candidate radiopharmaceuticals for further development or for theranostic applications.

This MIB Guide outlines a basic protocol for preclinical dosimetry estimation. The protocol is designed to strike a balance among rigor, prescriptiveness and simplicity, to thereby ensure it can effectively support the majority of preclinical dosimetry assessments while also being adaptable by investigators in the radiopharmaceutical sciences who may not have extensive medical physics backgrounds.

## OBJECTIVES

Use of this protocol should enable the investigator to appropriately utilize dosimetry software to derive organ-level dosimetry estimates from animal biodistribution data obtained empirical studies, literature, or other sources.

## THEORETICAL BACKGROUND

The basic protocol described is for organ-level dosimetry, and utilizes the mathematical framework of the MIRD schema^1,2^. Organ-level dosimetry typically calculates the mean absorbed dose delivered to target regions (i.e., organs or tissues where radiation energy is imparted) from estimates of the number of nuclear transformations that occur within source regions (i.e., organs or tissues containing a radionuclide) after administration of the radiopharmaceutical. The reader is strongly encouraged to refer to the MIRD Primer^1^ for a detailed examination of the fundamental principles of this schema. The essential dosimetric quantities of absorbed dose and effective dose are defined below.

### Absorbed dose

The absorbed dose $D\left(r_{T},T_{D}\right)$ [Gy] is the mean energy [J] imparted to a target region $r_{T}$ per unit tissue mass [kg], where the region is irradiated over a dose integration period $T_{D}[s]$. The absorbed dose coefficient $d\left(r_{T},T_{D}\right)[Gy/Bq]$ is the absorbed dose normalized to the administered activity $A_{0}\;[Bq]$. If the subject’s anatomy does not change with time, the absorbed dose coefficient can be calculated as:

Eqn. 1 $$d\left(r_{T},T_{D}\right)=\sum_{r_{S}}\;\tilde{a}\left(r_{S},T_{D}\right)S\left(r_{T}\leftarrow r_{S}\right)$$

where $\tilde{a}\left(r_{S},T_{D}\right)$ [s] is the time-integrated activity coefficient (TIAC) and $S\left(r_{T}\leftarrow r_{S}\right)$ [Gy/s.Bq], known as the ‘S-value’ (or S-coefficient), is the absorbed dose rate to target region $r_{T}$ per unit activity in source region $r_{S}$. Source regions and target regions refer to areas of the body within which nuclear transformations occur, and, where radiation energy is imparted, respectively; these may include whole organs, organ substructures, or groups of organs. The TIAC represents the number of nuclear transformations occurring in a source region over the dose integration period $T_{D}[s]$ normalized to the administered activity and can be computed as:

Eqn. 2 $$\tilde{a}\left(r_{S},T_{D}\right)=\int_{0}^{T_{D}}\;a\left(r_{S},t\right)dt$$

where $a\left(r_{S},t\right)$ is the administered activity-normalized activity in $r_{S}$ at time t post-administration. For radiopharmaceutical dosimetry, the dose integration period is usually taken to be infinity (i.e., to complete decay of the administered activity).

### Effective dose, equivalent dose, and RBE-weighted dose

The effective dose [Sv] is used as a single-value metric of the risk (i.e., the age- and gender-averaged risk) of radiation-induced stochastic effects in reference humans. It is a weighted sum of the equivalent doses to various organs and tissues of the human body, where the weighting factors reflect the relative sensitivity of each tissue to radiation-induced cancer or germ-cell mutagnesis^3(p107)^. The effective dose coefficient $e\left(T_{D}\right)[Sv/Bq]$ (i.e., effective dose per unit administered activity) is calculated as:

Eqn. 3 $$e\left(T_{D}\right)=\sum_{T}\;\frac{w_{T}h{\left(r_{T},T_{D}\right)}^{\text{Male}}+w_{T}h{\left(r_{T},T_{D}\right)}^{\text{Female}}}{2}$$

where $w_{T}$ is the tissue weighting factor for tissue T and $h\left(r_{T},T_{D}\right)$ [Sv] is the equivalent dose, wherein the radiation weighting factor $w_{R}$ accounts for biological effectiveness of each radiation type R for stochastic endpoints:

Eqn. 4 $$h\left(r_{T},T_{D}\right)=\sum_{R}\;w_{R}d_{R}\left(r_{T},T_{D}\right)$$

For considering deterministic effects such as therapy (i.e., tumor cell kill/sterilization) or tissue reactions (e.g., organ toxicity), the absorbed dose can also be weighted by an appropriate relative biological effectiveness (RBE) factor for different radiation types:

Eqn. 5 $$d_{RBE\text{-}weighted}\left(r_{T},T_{D}\right)=\sum_{R}\;RBE_{R}d_{R}\left(r_{T},T_{D}\right)$$

This utilization of RBE parallels the concept of the radiation weighting factor used in Eqn. 4. However, in the case of deterministic effects, the RBE-weighting factor relates to deterministic endpoints assessed under a set of specific experimental conditions, in contrast to a single set of consensus RBE values chosen by committee review for stochastic endpoints.^2^ There is no current consensus on the values of $RBE_{R}$ or the units for RBE-weighted dose for deterministic endpoints; for the latter, gray (Gy), gray-equivalent (Gy-Eq), or sievert (Sv) have been commonly used.

### Organ Level Dosimetry Software and Computational Phantoms

Numerous software tools are available to facilitate organ-level dosimetry computations in animal or human phantoms (Table 1). Most compute absorbed dose via Eqn. 1, using stored databases of S-values combined with user input of the TIAC for each source region of a computational reference phantom (a representative anatomical model). If biodistribution measurements have already been acquired, then the major remaining task is to compute the TIACs. This process is a central component of the protocol detailed in the proceeding section.

## BASIC PROTOCOL

### Strategy

**Figure 1:**
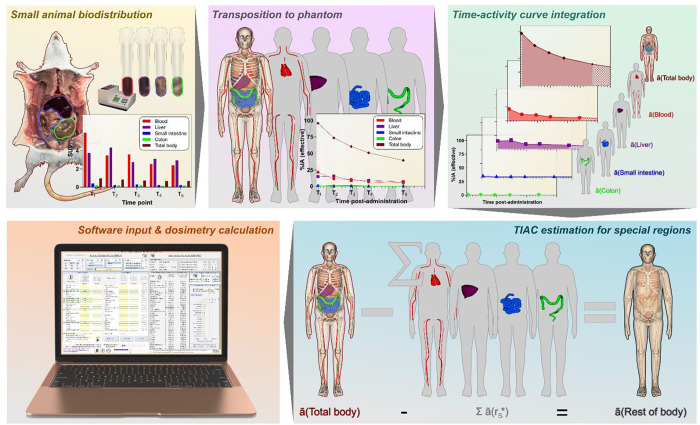
Dosimetry workflow summary.

The basic approach of this protocol is to obtain an organ-level dosimetry estimate starting from animal biodistribution data routinely quantified in units such as standard uptake value (SUV) or percent of the administered activity per gram (%IA/g) with the use of widely available organ-level dosimetry software tools which can perform curve-fitting and integration of time-activity data and the dose calculation (Fig. 1). Importantly, this protocol is predicated on the first-order assumption that the SUV is independent of body mass and species.

### Key Definitions

- Biological uptake or clearance: uptake or clearance of a radionuclide by biological processes that are independent of the physical decay of the radionuclide.
- Effective uptake or clearance: net uptake or clearance of a radionuclide by a combination of radioactive decay and biological processes.
- Standardized uptake value (SUV): a measure of activity concentration in an organ or tissue region, which is decay-corrected to time of administration and normalized to total body mass, that is, activity per unit mass of tissue divided by the activity administered per unit body mass.

### General Assumptions

- Subject anatomy (e.g., organ size and shape) is generally assumed to be time-invariant and is matched to a standard reference anatomic phantom used for the dosimetry calculations.
- The radionuclide is homogeneously distributed with the respective source regions (i.e., organs)
- The radionuclide’s emissions, in terms of their respective frequencies and energies, occur according to established decay schemes
- No radioactive progeny are generated by the decay of the radionuclide
- The biodistribution, quantified in units of SUV, is equivalent among species and subjects of different total-body masses and the computational phantoms used to represent them.
- Tissue harvested for biodistribution measurements include the tissue parenchyma and blood (i.e., blood has not been removed from harvested tissue).

### Step 1: Familiarization with Software and Phantom

Prior to performing biodistribution measurements, the investigator should familiarize themselves with the pertinent phantoms (i.e., anatomic models) and software that will be used in the dosimetry computation software to ensure the tissues to be assayed are consistent with the source-regions defined in the phantom and the software. Regardless of the software chosen, the investigator is strongly encouraged to thoroughly read its documentation and/or manual.

### Step 2: Biodistribution Measurement

Biodistribution measurements identify the regions of the body where activity accumulates and establish how the activity changes over time in these regions. There are several key study design considerations for biodistribution measurements: 1) What measurement technique will be used? 2) What regions will be analyzed? 3) At what time points post-administration will measurements be performed? 4) How many animals will be used?

In the preclinical setting, activity quantification is usually performed by either *ex vivo* radiometric counting (e.g., gamma counting, liquid scintillation counting) of tissues harvested from small animals or by segmentation of serially acquired small-animal PET, SPECT, or planar gamma camera images. *Ex vivo* counting is considered the ‘gold standard’ for organ-level dosimetry, primarily due to its high sensitivity, quantitative accuracy, simplicity, and reproducibility. Additionally, for small-animal imaging anesthesia is required at each measurement time point and may impact the data thus measured. Therefore, this Guide recommends *ex vivo* measurements. This approach, however, requires a larger number of animals, as a statistically reliable number of animals (typically at least 3) must be sacrificed at each measurement time point, and is generally more time-consuming and labor-intensive than imaging-based biodistribution studies.

#### Step 2a: Select organ and tissues to be analyzed

Selection of anatomic regions for analysis should be based on available data or an *a priori* understanding of the radiopharmaceutical, including organ(s) of likely highest uptake, excretion route, metabolism and likely biodistribution and excretion route of radiometabolites. At minimum, the source regions sampled should include those exhibiting the highest activity concentrations, radiosensitive tissues with demonstrable accumulation or retention, blood, contents of the major excretory pathways (e.g., urine, GI contents), and the whole body or animal carcass (to enable estimation of the total-body and rest-of-body activities). To ensure that all activity can be accounted for, it is also advisable to measure the activity present in the bedding material that contains the animal excreta.^17^ However, it is often difficult to impossible to quantitatively recover all of the activity administered unless animals are housed individually in metabolic cages, but this is rarely done in practice.

#### Step 2b: Select time points and study population size

An essential consideration in study design is the number and temporal spacing of the biodistribution samples. Ideally, biodistribution should be sampled at a sufficient number of time points which are temporally spaced such that the uncertainty in the TIAC is minimized (see Step 4); this can usually be accomplished by ensuring that each component of uptake and clearance can be identified. Since various source regions may exhibit distinct uptake and clearance patterns, it is challenging to determine time points that are simultaneously optimal for all such regions, especially in light of logistical considerations such as the rapid decay of short-lived radionuclides, cost, and limitations on animal cohort size. The ICRU has proposed that time points be selected based on multiples of the effective half-life, $T_{e}$, in the blood or other source region of interest - namely, $T_{e}/3,\;2T_{e}/3,\;3T_{e}/2,\;3T_{e}$, and $5T_{e}{\;}^{18(p16),19}$ Based on our experience, we propose the following empirical function for roughly determining an appropriate sequence of time points:

Eqn. 6 $$T_{i}=T_{min}+\left(T_{max}-T_{min}\right)\cdot {\left(\frac{i-1}{n-1}\right)}^{1.5}\text{for}\;\text{time}\;\text{point}\;i=1,2,\ldots ,n\;T_{min}=\frac{T_{e}}{n-1}\;T_{max}=\frac{1.5\cdot n\cdot T_{e}}{0.15\cdot n+1}$$

where n is the number of measurement time points, $T_{i}$ is the time post-administration of measurement i, and $T_{min}$ and $T_{max}$ are the times post-administration of the first and last (i.e., the earliest and the latest) measurements, respectively.

Using a larger sample size will typically increase the precision of the dosimetry estimates. Realistically, the number of time points and the number of animals per time point should be chosen based on several factors such as the research question, resources available, animal welfare considerations, and the expected variability in the data. For dosimetry purposes, it is generally preferable to obtain fewer replicates (i.e., animals) at more time points rather than vice versa.

A minimum of 4 time points is recommended for adequate temporal resolution of the time-activity curve. A minimum of 3–4 animals per time point is recommended for adequate statistical power for the tissue activity measurements. For statistical rigor in curve-fitting of time-activity data, a degree of freedom (i.e., the number of measurements minus the number of parameters of the curve) of at least 1 is required. For example, for a bi-exponential curve, with 2 zero-time intercepts and 2 clearance constants and therefore a total of 4 fittable parameters, at least 5 time points would be required. In practice, however, such statistical rigor is often sacrificed.

#### Step 2c: Quantify activity concentration in units of SUV

The SUV is a measure of activity concentration in a region, which is decay-corrected to the time of administration and normalized to total body mass:

Eqn. 7 $$SUV{\left(r_{S}^{AN}\right)}_{i,j}=[\%IA/g]{\left(r_{S}^{AN}\right)}_{i,j}\cdot \frac{M{\left({\text{Total}\;\text{body}}_{S}^{AN}\right)}_{i,j}}{100}$$

where for animal source region $r_{S}^{AN}$ the index i represents a time point, index j represents a replicate at that time point, and $M\;({Total\;body\;}_{S}^{AN})$ is the animal mass. The bracketed quantity, %IA/g, is the activity mass concentration in units of percentage of administered activity per gram of tissue, decay-corrected to the time of administration. Of note, %IA/g is also commonly also referred to as %ID/g or %AA/g, where “ID” and “AA” are the injected dose and the administered activity, respectively. The superscript “AN” is an abbreviation denoting that the quantity is for the specific animal subject; the notations “HU” and “PH” will be used to denote quantities specific to a human or a phantom, respectively. Importantly, if the activity concentration measurements quantify volume concentration (as is usually the case for tomographic images), they must be divided by the respective tissue density (often assumed to be 1 g/cm^3^ for soft tissues) to obtain the mass concentration.

Finally, the mean SUV among the animal subjects at each time point is computed. If the animal body masses are reasonably consistent across the study population, the mean SUV can be approximated using the animal population mean body mass, $\overset{‾}{M}\;({Total\;body\;}_{S}^{AN})$:

Eqn. 8 $$\bar{SUV}{\left(r_{S}^{AN}\right)}_{i}\approx \bar{\left[\%IA/g\right]}{\left(r_{S}^{AN}\right)}_{i}\cdot \frac{\overset{‾}{M}({\text{Total}\;\text{body}}_{S}^{AN})}{100}$$

Standard deviations of the mean values of the respective activity concentration parameters at each time point should recorded, not only as an indicator of the “quality” of the time-activity data but also because curve-fitting software often weight the data points by their respective standard deviations, fractional standard deviations, or other metrics of the statistical reliability of the data points.

### Step 3: Transpose Biodistribution into Phantom Source Regions

Organ-level dosimetry software typically requires input of the TIAC for source regions of a reference phantom. The phantom may represent the same species as the study subject(s) or a different species. Importantly, dosimetry estimates computed using a reference phantom involve extrapolation, that is, the biodistribution for the phantom is derived from the study subject(s) which differ in anatomy from the phantom.

Several methods have been introduced for such extrapolations. The first-order approach prescribed by this Guide assumes that the body mass-normalized SUV is equivalent among species and phantoms used to approximate them:

Eqn. 9 $$\bar{SUV}{\left(r_{S}^{AN}\right)}_{i}=\bar{SUV}{\left(r_{S}^{HU}\right)}_{i}=\bar{SUV}{\left(r_{S}^{PH}\right)}_{i}\to \bar{SUV}{\left(r_{S}\right)}_{i}$$

The fraction of administered activity in the phantom source regions, $a\left(r_{S},t\right)$, is required to apply Eqn. 2 in the next step of this protocol; importantly, $a\left(r_{S},t\right)$ must not be decay-corrected. This quantity can be computed by scaling the SUV for source region $r_{S}$ with its corresponding mass in the phantom, *viz*:

Eqn. 10 $$a{\left(r_{S}^{PH}\right)}_{i}=\bar{SUV}{\left(r_{S}\right)}_{i}\cdot \frac{M\left(r_{S}^{PH}\right)}{M\left({\text{Total}\;\text{body}}_{S}^{PH}\right)}\cdot e^{-\lambda_{p}T_{i}}$$

where $M\left(r_{S}^{PH}\right)$ is the mass of $r_{S}$ defined in the phantom, $M\;(Total\;body\;{\;}_{S}^{PH})$ is the total phantom body mass, $\lambda_{p}$ is the physical half-life of the radionuclide, $T_{i}$ is the time post-administration of the $i^{\text{th}}$ time point, and the exponential term negates the decay correction inherent in the definition of SUV. This scaling approach obviates the need for knowledge of the masses of the harvested animal tissue samples (assuming the SUV or %IA/g are known) but requires knowledge of the phantom source-region masses. Importantly, the source region masses should be inclusive of blood within the regions since the harvested tissue samples include blood. The phantom source-region masses can typically be found within the dosimetry software to be used.

#### Step 3a: Estimate fraction of administered activity in standard source regions

Application of Eqn. 10 is generally straightforward for the source regions corresponding to the major visceral organs (i.e., ‘standard’ source regions). Some noteworthy exceptions are discussed below.

#### Step 3b: Estimate fraction of administered activity in the red bone marrow source region

The hematopoietic (red) marrow is extremely radiosensitive and is commonly the dose-limiting tissue for therapeutic radiopharmaceuticals. It is difficult to harvest pure red marrow samples from the bones of small-animal subjects. For dosimetric purposes, it is commonly assumed that the activity concentration in the blood is representative of that of the red bone marrow; implicit in this is the assumption that the administered radiopharmaceutical does not localize specifically in or on any marrow component. This is typically a conservative assumption reasonable for translational dosimetry. Therefore, the protocol adopts this assumption and prescribes the following equation for computing the TIAC of phantom red marrow:

Eqn. 11 $$a(\text{Red}\;\text{marrow}{)}_{i}=\bar{SUV}{\left({Blood}_{S}\right)}_{i}\cdot \frac{M({\text{Red}\;\text{marrow}}_{S}^{PH})}{M\left({\text{Total}\;\text{body}}_{S}^{PH}\right)}\cdot e^{-\lambda_{p}T_{i}}$$

where $M\;{(\text{Red}\;\text{marrow}}_{S}^{PH})$ is the mass of the phantom red-marrow source region.

#### Step 3c: Estimate total body fraction of administered activity in the phantom

The fraction of administered activity in the whole body can be used to determine the TIAC for the urinary bladder and the rest-of-body source regions. Due to differences in experimental design and measurement capabilities, we have provided several options for computation of the total body TIAC. If the animal total-body mean activity concentration is known, then the $a(\text{Total}\;\text{body}{)}_{i}$ can be calculated via Eqn. 8–10:

Eqn. 12 $$a(\text{Total}\;\text{body}{)}_{i}=\bar{SUV}{\left({\;\text{Total}\;\text{body}\;}_{S}\right)}_{i}\cdot e^{-\lambda_{p}T_{i}}\;\approx \bar{\left[\text{\%}IA/g\right]}{\left({\text{Total}\;\text{body}}_{S}^{\text{AN}}\right)}_{i}\cdot \frac{\bar{M}\left({\text{Total}\;\text{body}}_{S}^{\text{AN}}\right)}{100}\cdot e^{-\lambda_{p}T_{i}}$$

Eqn. 12 is generally useful for deriving $a(\text{Total}\;\text{body}{)}_{i}$ from animal image data using a region of interest encompassing the entire body of the animal or from *ex vivo* measurements of the intact animal.

If mean activity concentration is estimated from animal carcass measurements, as is common is *ex vivo* counting experiments, then the $a(\text{Total}\;\text{body}{)}_{i}$ may be calculated by summing the contribution of the carcass tissue with the contributions of tissues that have been removed from it. However, care must be taken not to ‘double count’ tissues that have been *partially* sampled from the carcass:

Eqn. 13 $$a(\text{Total}\;\text{body}{)}_{i}=(\sum_{r_{S}\in \text{Carcass}}\;\bar{SUV}{\left({\text{Carcass}}_{S}\right)}_{i}\cdot \frac{M\left({\text{Carcass}}_{S}^{PH}\right)}{M({\text{Total}\;\text{body}}_{S}^{PH})}+\sum_{r_{S}\notin \text{Carcass}}\;\bar{SUV}{\left(r_{S}\right)}_{i}\cdot \frac{M(r_{S}^{PH})}{M({\text{Total}\;\text{body}}_{S}^{PH})})\cdot e^{-\lambda_{p}T_{i}}$$

Eqn. 13 is generally useful for deriving $a(\text{Total}\;\text{body}{)}_{i}$ from *ex vivo* biodistribution measurements where the activity concentrations are available for animal organs and remaining carcass, but the masses of the tissues are not.

Assay of whole-body activities in mice is challenging, as a mouse carcass may not fit in a typical (i.e., 20 mL) counting vial. Another approach to assaying whole-body activity is as follows. Briefly place the anesthetized animal, in a plastic bag, in a dose calibrator *immediately* post-radiopharmaceutical administration and record the activity reading $A\left(T_{0}\right)$. Although this activity reading is not accurate (as dose calibrators are not calibrated for sources as irregularly sized and shaped as mice), the activity reading $A\left(T_{0}\right)$ corresponds to 100% of the administered activity and any subsequent dose-calibrator activity reading for the animal at various measurement time points $T_{i},\;A\left(T_{i}\right)$, may be divided by $A\left(T_{0}\right)$ to yield the fraction of the administered activity remaining in the body at time $T_{i}$. The rest-of-body activity at each time point can then be calculated as the difference between the total-body activity thus determined and the sum of the respective organ activities determined by *ex vivo* counting (see Eqn. 18 below).

### Step 4: Time-Activity Curve Integration

At this point, we have estimated fractions of administered activity in the phantom source regions at discrete time points on a continuous time-activity curve, namely, $a\left(r_{S},t\right)$ versus t. For each source region, Eqn. 2 must be applied to compute the respective TIAC needed as input in organ-level dosimetry software. Two approaches have frequently been used for evaluating the integral in Eqn. 2: 1) trapezoidal integration and 2) exponential regression and analytic integration.

#### Trapezoidal integration

In this variation, the time-activity curve is approximated as a piecewise linear function that passes through each measured point. The area under this function is then computed using the trapezoidal rule, wherein the area is divided into trapezoids with vertices defined by the measured points and their projections onto the time axis. The trapezoidal areas are then summed to determine the TIAC accumulated over the experimental measurement interval (i.e., between the first and last measured time points, $T_{1}$ to $T_{n}$). Finally, the contributions to the TIAC must be estimated for the intervals spanning the time of injection to first measured time point, (t=0 to $T_{1}$) and beyond the last measured time point ($T_{n}$ to ∞); the latter, in particular, may represent a substantial portion of the total time integral. To estimate these contributions, two assumptions are commonly made, respectively: 1) the fraction of administered activity at the time of injection is approximately equal to that at the first time point, and 2) conservatively, the activity is eliminated by physical decay only following the last time point. Given those assumptions, the total TIAC can be computed as:

Eqn. 14 $$\tilde{a}\left(r_{S},T_{D}\right)=\int_{0}^{T_{1}}\;a\left(r_{S},t\right)\;dt+\int_{T_{1}}^{T_{n}}\;a\left(r_{S},t\right)\;dt+\int_{T_{n}}^{T_{D}=\infty }\;a\left(r_{S},t\right)\;dt\approx T_{1}\cdot a{\left(r_{S}\right)}_{1}+\left[\sum_{i=1}^{n-1}\;\frac{a{\left(r_{S}\right)}_{i+1}+a{\left(r_{S}\right)}_{i}}{2}\left(T_{i+1}-T_{i}\right)\right]+\frac{a{\left(r_{S}\right)}_{n}}{\lambda_{p}}$$

Alternate forms for the first and last terms of Eqn. 14 based on different assumptions, with justification, may be used.

#### Exponential regression

In this variation, a function comprising one or more exponential terms is fit to the measured points using the method of nonlinear least-squares regression. This approach involves minimizing the sum of squared differences between predicted and measured values of the fraction of administered activity, by adjusting the function’s parameters until the optimal (i.e., best fit) parameter values are found. The general form of fitting functions used for radiopharmaceutical dosimetry is:

Eqn. 15 $$a\left(r_{S},t\right)=\sum_{\psi =1}^{\Psi \left(r_{S}\right)}\;c_{\psi }\left(r_{S}\right)e^{-\left[\lambda_{b\psi }\left(r_{S}\right)+\lambda_{p}\right]t}$$

where $\lambda_{b\psi }$ are biological rate constants for different components of clearance denoted by the index ψ; the exponential amplitude coefficients (i.e., the zero-time values) for each component are given by $c_{\psi }$; and $\lambda_{p}$ is the physical decay constant. Once the best fit parameter values are found, the analytical expression for the TIAC (obtained by substituting Eqn. 15 into Eqn. 2) can be evaluated:

Eqn. 16 $$\tilde{a}\left(r_{S},T_{D}\right)=\int_{0}^{T_{D}=\infty }\;\sum_{\psi =1}^{\Psi \left(r_{S}\right)}\;c_{\psi }e^{-\left[\lambda_{b\psi }\left(r_{S}\right)+\lambda_{p}\right]t}dt=\frac{c_{1}\left(r_{S}\right)}{\lambda_{b1}\left(r_{S}\right)+\lambda_{p}}+\frac{c_{2}\left(r_{S}\right)}{\lambda_{b2}\left(r_{S}\right)+\lambda_{p}}+\cdots +\frac{c_{\Psi }\left(r_{S}\right)}{\lambda_{b\Psi }\left(r_{S}\right)+\lambda_{p}}$$

The total number of components of clearance, Ψ, is either assumed by the investigator or may be estimated by objectively comparing fit of multiple exponential models based on metrics for goodness of the fit (e.g., Akaike information criterion)^20^; the latter is outside the scope of this guide. Importantly, Ψ should never be chosen such that the total number of adjustable parameters exceeds the number of time points minus 1; this latter quantity is known as the degrees of freedom, as previously noted.

If the amplitude coefficients of the function in Eqn. 15 are constrained to positive values, then Eqn. 15 effectively ignores the uptake, or “rising,” portion of the time-activity curve. Generally, this portion of the curve is fairly brief and contributes relatively little to the total TIAC and therefore may be ignored for purposes of dosimetry. However, if this is not the case, the aforementioned constraint may be removed, or, a more complex function than that in Eqn. 15 may be used; for such cases, the reader is referred to Section 5.7.1 of the MIRD Primer 2022.^1^

Several software tools are designed for performing exponential regression for radiopharmaceutical dosimetry, including MIRDfit^21^,NUKFIT^20^, and OLINDA/EXM. Other more general statistical software also may be used; these include SAAM-II^22^, Graph Pad Prism, and the Microsoft Excel Solver add-in.

#### Step 4a: Compute the TIACS for the standard source regions

For source regions corresponding to major visceral organs, compute the TIAC using Eqn. 16 or Eqn. 14.

#### Step 4b: Determine the total-body clearance parameters and TIAC using exponential regression

The clearance parameters for the whole body can be used to estimate the TIAC for unique regions of the body, including the urinary bladder and the rest of body (*vide infra*).

Determine the biological clearance constants for each component of clearance $\left(\lambda_{b1},\lambda_{b2},\ldots \right)$ and the amplitudes $\left(c_{1},c_{2},\ldots \right)$ for the total-body time-activity curve by regression using Eqn. 15. Next, compute the TIAC for the total body via Eqn. 16; this will be needed for the subsequent step.

In case there is no biological elimination of activity, the TIAC for the total body is equivalent to the mean lifetime of the radionuclide, $1/\lambda_{p}$; in such cases, Step 4c should be skipped.

#### Step 4c: Compute the TIAC for the urinary bladder via the voiding bladder excretory model (if urinary excretion is significant)

The rapid filling and voiding processes of the urinary bladder conflict with the assumption of time-invariant organ size. Filling and voiding may occur repeatedly among time point measurements, confounding assessment of the time-activity curve. Moreover, quantitative collection of urine from small animals often presents challenges (e.g., sample loss due to micturition during euthanasia). An alternative to direct measurement of the urinary bladder TIAC has been demonstrated, which estimates the urinary bladder TIAC based on total body clearance^23,24^:

Eqn. 17 $$\tilde{a}(Bladder,\left.T_{V}\right)=\sum_{\psi =1}^{\Psi \left({\text{Total}\;\text{body}}_{S}\right)}\;f_{u\psi }\left(\frac{1-e^{-\lambda_{p}T_{V}}}{\lambda_{p}}-\frac{1-e^{-\left[\lambda_{b\psi }\left({\text{Total}\;\text{body}}_{S}\right)+\lambda_{p}\right]T_{V}}}{\lambda_{b\psi }\left({\text{Total}\;\text{body}}_{S}\right)+\lambda_{p}}\right)\left(\frac{1}{1-e^{{-\left[\lambda_{b\psi }\left({Total\;body}_{S}\right)+\lambda_{p}\right]T}_{V}}}\right)$$

where $f_{u\psi }$ is the fraction of total biological excretion that occurs via the $\psi^{\text{th}}$ component of urinary excretion, and $T_{V}$ is the bladder voiding interval.

#### Step 4d: Compute the TIAC for the rest of body

The rest of body is a unique source region representing the collective tissues that have not been explicitly assigned a TIAC. The dose contribution from the rest-of-body TIAC can be estimated with dosimetry software (Step 5).

The TIAC for the phantom rest of body, $\tilde{a}$ (Rest of body), can be computed as:

Eqn. 18 $$\tilde{a}(Rest\;of\;body)=\tilde{a}(Total\;body)-\sum_{r_{S}*}\;\tilde{a}\left(r_{S}{\;}^{*}\right)$$

where $r_{S}{\;}^{*}$ is a source region for which a TIAC was explicitly derived.

### Step 5: Calculate dosimetry via Software

**Figure 2:**
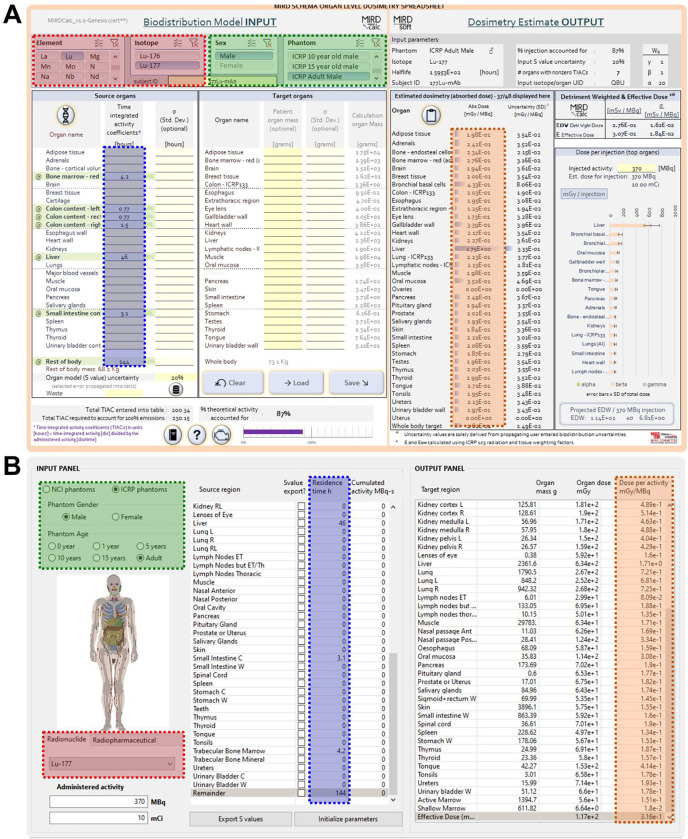
Calculation input for two typical organ-level dosimetry softwares. A) MIRDcalc software. B) NCINM software. The shaded boxes indicate the required user inputs: red – radionuclide; green – phantom; blue – TIAC. The boxes with orange shading indicate the absorbed doses computed.

The final step of this protocol is to configure the dosimetry calculation in the organ-level dosimetry software of choice. In most software, this is intuitive and requires minimal explanation: 1) Select radionuclide, 2) select anatomic phantom, 3) Enter TIACs^†^ for the source regions (Fig. 2). As a critical note, the investigator must ensure the units of the input TIACs are in the units expected by the software; typically, units of hours are used. If supported by the software, the investigator may enter RBE-weighting values for the different radiation types, which may be of particular use for alpha- or Auger electron-emitting radiopharmaceuticals.

Following input of the TIACs, most software will immediately report: 1) the absorbed dose coefficients *or* equivalent dose coefficients for each target organ and, 2) the effective dose coefficient.

### Step 6: Examine results

Naturally, every computation should undergo a “sanity” check to judge the reliability of the results. The results should be checked for consistency; that is, it should be verified that the dose coefficients are within the expected range based on previous studies or reference values^25(p128)^ for similar radiopharmaceuticals. If major discrepancies are identified, their source(s) should be investigated. Most software will generate an input echo to aid in identifying potentially mis-entered data or other blunders. Cross-validation may also be useful – namely, using alternative dosimetry models, TAC fitting approaches, or software tools to independently calculate the dosimetric quantities for comparison. One approach to checking dosimetry results is based on the reasonable assumption that the major dose contribution to a particular tissue is due to complete local absorption of particulate-radiations (i.e., beta particles, electrons, and/or alpha particles) emitted within the tissue. This tissue “self dose” may then be approximated as the product of the tissue’s TIAC per unit mass and the radionuclide’s mean energy emitted per decay in the form of such non-penetrating (np) radiations, *Δ*_*np*_, available in various tabulations of nuclear decay-scheme data. This estimate of each tissue’s absorbed dose coefficient and that reported by the dosimetry software should be in reasonable, though not exact, agreement.

## PROTOCOL IN ACTION: A WORKED EXAMPLE

A worked and annotated example of a human dosimetry estimate extrapolated from murine biodistribution data is provided in the electronic supplemental information.

## CONCLUSION

A basic protocol for preclinical dosimetry estimation is provided. It is intended to serve as a resource for deriving dosimetry estimates to support basic and translational radiopharmaceutical science. Although the concepts discussed can be applied to dosimetry in human research subjects or patients, the clinical application of these concepts should involve trained personnel, including a nuclear medicine physician and qualified medical physicist, as well as the use of approved software.

## Figures and Tables

**Table 1:** Organ-level dosimetry software.

Software	Phantoms	Accessibility
IDAC-Dose^4^	ICRP 110 reference adult^5(p110)^	Free
MIRDcalc^6^	ICRP 110 reference adult^5^ ICRP 143 reference pediatric^7^ Interpolated human reference phantoms	Free
NCINM^8^	UF-NCI reference adult^9^ UF-NCI reference pediatric^9^ ICRP 110 reference adult^5^ ICRP 143 reference pediatric^7^	Free
OLINDA 1.0	ORNL reference adult^10^ ORNL reference pediatric^10^	Discontinued
OLINDA 2.0^11^	RADAR reference adult RADAR reference pediatric^12^ RADAR pregnant female^12^ ORNL reference adult^10^ ORNL reference pediatric^10^ ONRL pregnant female^10^ ROBY reference rat^13^ MOBY reference mouse^13^ Rusty/Zena dog	Commercial
PARaDIM^14^	Digimouse (mouse)^15^ Various other phantoms supported	Requires PHITS^16^ (free)

## CHALLENGING STEP

If urinary excretion is the only excretion route, then the urinary clearance fraction(s) $f_{u\psi }$ are simply the total body clearance components $c_{\psi }$.

If activity is excreted through a combination of hepatobiliary and urinary pathways, isolation of the urinary clearance components becomes more complex. In first order, $f_{u\psi }$ may be estimated based on the half-lives for biological excretion (i.e., $T_{b\psi }=ln(2)/\lambda_{b\psi }$) of activity from the whole body. For example, if the half-life for urinary excretion is assumed to be short - on the order of several hours or less, excretion components with biological half-lives longer than this time period may be reasonably excluded from Eqn. 17. Another approach involves exclusion of the activity in the intestines (i.e., resultant from Gl excretion) prior to fitting the total body time-activity curve.^24^ Neither approach is rigorous, but either is often adequate for the purposes of preclinical dosimetry. If metabolic caging is utilized, urinary excretion can be more rigorously characterized by fitting a function of the form $\sum_{\psi =1}^{\Psi }\;c_{\psi }\left(1-e^{-\lambda_{b\psi }\cdot t}\right)$ to cumulative biological urinary excretion followed by application of Eqn. 17.

## Footnotes

† The *time-integrated activity coefficient* is also commonly referred to by equivalent terms which may be displayed by different softwares. Such terms include the *residence time*, *number of disintegrations*, and *normalized cumulated activity*; all are equivalent provided they are expressed in equivalent units of time (e.g., hours, MBq·h/MBq).
